# Projection of Diabetes Population Size and Associated Economic Burden through 2030 in Iran: Evidence from Micro-Simulation Markov Model and Bayesian Meta-Analysis

**DOI:** 10.1371/journal.pone.0132505

**Published:** 2015-07-22

**Authors:** Mehdi Javanbakht, Atefeh Mashayekhi, Hamid R. Baradaran, AliAkbar Haghdoost, Ashkan Afshin

**Affiliations:** 1 Health Economics Research Unit, Institute of Applied Health Sciences, University of Aberdeen, Aberdeen, United Kingdom; 2 Endocrine Research Center, Institute of Endocrinology & Metabolism, Iran University of Medical Sciences, Tehran, Iran; 3 Endocrine Research Center, Institute of Endocrinology & Metabolism, Iran University of Medical Sciences, Tehran, Iran; 4 Kerman University of Medical Sciences, Kerman, Iran; 5 Friedman School of Nutrition Science and Policy, Tufts University, Boston, United States of America; Medical College of Soochow University, CHINA

## Abstract

**Background:**

The aim of this study was to estimate the economic burden of diabetes mellitus (DM) in Iran from 2009 to 2030.

**Methods:**

A Markov micro-simulation (MM) model was developed to predict the DM population size and associated economic burden. Age- and sex-specific prevalence and incidence of diagnosed and undiagnosed DM were derived from national health surveys. A systematic review was performed to identify the cost of diabetes in Iran and the mean annual direct and indirect costs of patients with DM were estimated using a random-effect Bayesian meta-analysis. Face, internal, cross and predictive validity of the MM model were assessed by consulting an expert group, performing sensitivity analysis (SA) and comparing model results with published literature and national survey reports. Sensitivity analysis was also performed to explore the effect of uncertainty in the model.

**Results:**

We estimated 3.78 million cases of DM (2.74 million diagnosed and 1.04 million undiagnosed) in Iran in 2009. This number is expected to rise to 9.24 million cases (6.73 million diagnosed and 2.50 million undiagnosed) by 2030. The mean annual direct and indirect costs of patients with DM in 2009 were US$ 556 (*posterior* standard deviation, 221) and US$ 689 (619), respectively. Total estimated annual cost of DM was $3.64 (2009 US$) billion (including US$1.71 billion direct and US$1.93 billion indirect costs) in 2009 and is predicted to increase to $9.0 (in 2009 US$) billion (including US$4.2 billion direct and US$4.8 billion indirect costs) by 2030.

**Conclusions:**

The economic burden of DM in Iran is predicted to increase markedly in the coming decades. Identification and implementation of effective strategies to prevent and manage DM should be considered as a public health priority.

## Introduction

Diabetes mellitus (DM) is among the leading causes of mortality worldwide, with an estimated at least 1.3 million deaths attributed to the illness in 2013 alone [1]. In 2013, an estimated 382 million people lived with DM worldwide and current projections suggest this number will rise to 592 million by 2035 [2,3]. Continuously increasing length of the life span of individuals in combination with the growing number of the world population are two underlying factors to the expected explosion in the numbers of diabetic patients and related burden.

Diabetes mellitus is a risk factor for other chronic health conditions, such as cardiovascular disease and complications resulting from it include nephropathy, amputations and blindness, all of which impose a burden to society by reducing quality of life, increasing the risk of pre-mature death and raising the economic burden due to absenteeism in the labor market and increased health care costs [4–10]. The estimated worldwide cost of DM and its associated complications was estimated to be at least US$548 billion in 2013[3].

The Middle East and North Africa (MENA) region has the highest diabetes prevalence in the world at 10.9%. It is estimated that about 35 million people are living with diabetes in this region [3]. Iran is amongst the countries with the highest prevalence of DM in the region at 9.94% in the adult population [3]. It has been suggested that socioeconomic development and urbanization have led to changes in lifestyle, such as increased sedentary activity and caloric intake coupled with a loss of traditional healthy dietary habits, which are responsible for the observed rise [11,12]. Furthermore, unlike most developed countries, where approximately half of reported cases are individuals older than 60 years, DM is most prevalent amongst the working population (20–59 years old), making it a major obstacle toward economic growth in Iran and other countries in the MENA region [13–15].

With rising health care costs and limited resources, it is necessary to understand the impact of DM in Iran to inform health policy and health care resource allocation. However, few studies have investigated the economic burden of DM in Iran and even less studies have estimated projections of the economic burden [16–18]. Given that a well-designed and validated model can effectively synthesize and combine data from various sources to generate new insights into the impact of chronic disease on society and reveal important gaps in the knowledge [19,20], therefore we developed this study which is the first study to use a Markov-microsimulation model to estimate the economic burden of DM in Iran from 2009 to 2030 using local database.

## Methods

### Model framework

A Markov microsimulation (MM) model was constructed to predict the growth of DM within the Iranian population over 22 years and its associated economic burden. The MM model is a computer modelling technique that simulates individual lives. Within the model each person is represented by a record containing a unique identifier and a set of associated attributes e.g. age, sex, disease condition, etc. A set of rules (transition probabilities) and states reward (cost and health state utility) are then applied to these characteristics. These rules may be deterministic or stochastic. The model applies all the defined parameters and rules over many time periods and allows the passage of individuals through the model one at time, generating individual life histories of a specified population [21,22].

**Fig 1:**
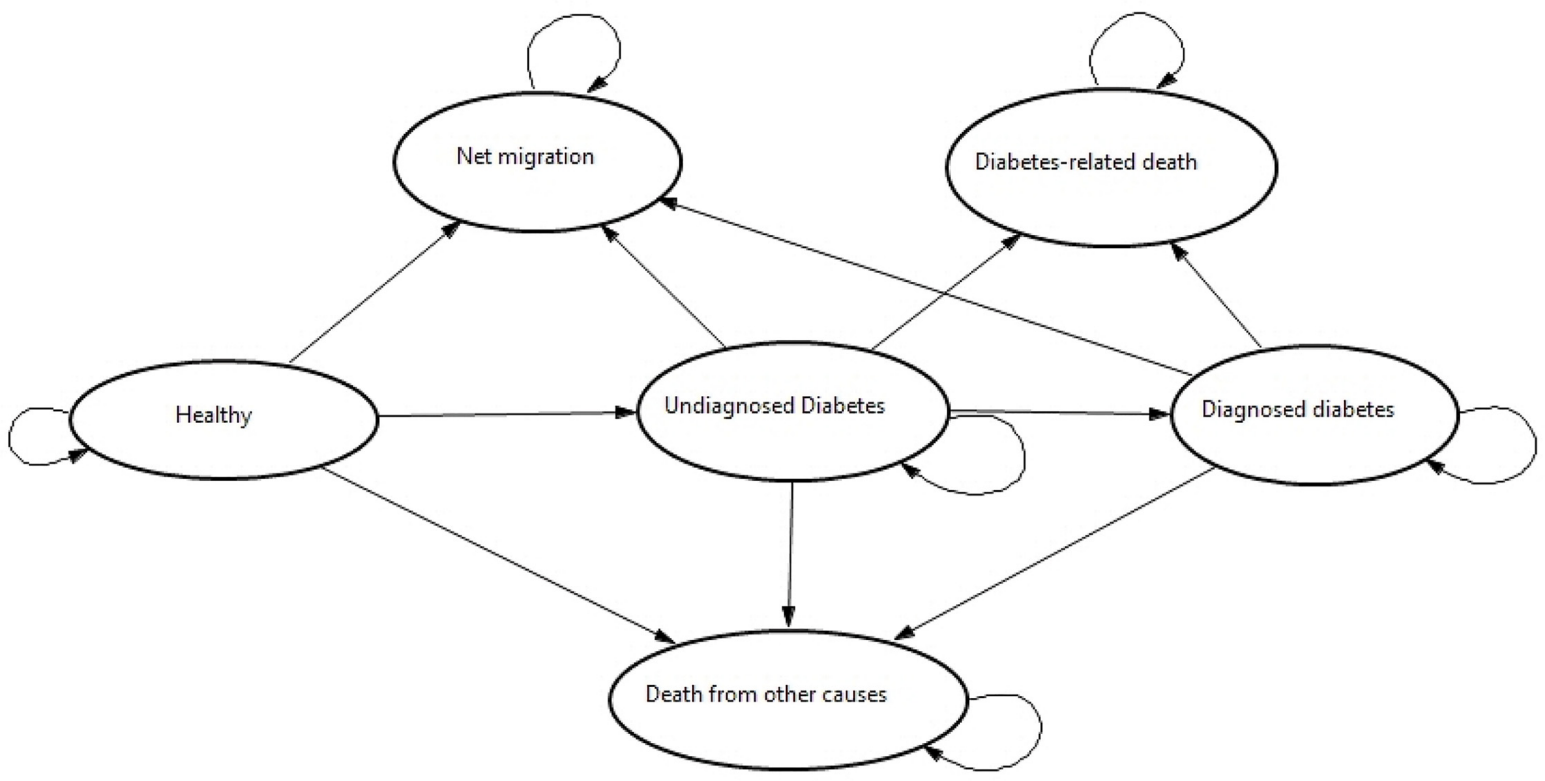
Markov model structure.

Demographic information (from national census), diabetes epidemiological data (from national Surveillance of Risk Factors of Non-Communicable Diseases (SuRFNCD[23]) and Tehran Lipid and Glucose Study (TLGS) [24]), and other economic data [16–18,25,26] were used to populate the model. To estimate the total burden of DM, a cohort using a representative sample of both women and men from 2009 was used (36,300 female and 37,400 male, which constitutes one thousandth of the total population of Iran) in the simulation. The MM model contained six health states: Healthy, Undiagnosed diabetes, Diagnosed diabetes, Net migration, Diabetes-related death and Death from background mortality (Fig 1). The arrows between the Markov states represent the possibility that an individual can remain in that specific Markov state for the next cycle. The expected consequences were estimated using a 22 year time-horizon (2009–2030). We assumed that transitions between health states occurred annually and their probabilities were derived from previous studies and national databases. Simulated patients passed through the model one at a time and exited through emigration, diabetes-related, or background death. If a case developed DM, the model tracked the disease progression in the cohort based on the defined transition probabilities.

### Data sources

#### Demographic inputs

The age distribution was derived from the national census data [27] and defined into the model as distribution. Iran’s 2009 life tables were used to estimate age- and sex-specific probabilities of death [28]. Diabetes-related input parameters were conditional on geographic location (i.e., urban or rural). Estimates for the percentage of the population living in urban areas was derived from the World Bank database[29]. Average annual net migration rate (per 1,000 people) was used to estimate immigration/emigration within the population. The net migration rate was derived from Department of Economic and Social Affairs of the United Nation [30].

### Diabetes epidemiology inputs

The initial distribution of the simulated cohort in the Markov states was based on the age- and sex-specific prevalence of diagnosed and undiagnosed cases of diabetes, which was retrieved from the SuFRNCD. The SuRFNCD has provides demographic, anthropometric and biochemical characteristics on a nationally representative sample of Iranian adults. Data from the TLGS study was used to derive the age- and sex-specific incidence of type 2 DM (T2DM)[31]. Since TLGS is a cohort study on the Iranian urban population, extracted incidence rates were applied for the urban population only (69% of the total population) [29]. Fasting plasma glucose (FPG) levels were compared between rural and urban populations to create adjusted incidence rates. As the average FPG level is lower among rural compared to urban populations, we applied a hazard ratio of 3.30 (2.65–4.10) in men and 3.54 (2.94–4.26) in women to adjust the incidence rate of T2DM in urban population, which was then applied to the rural population [31]. Age- and sex-specific incidence rates for Type 1 DM (T1DM) in rural and urban areas were derived from the study conducted by Pishdad et al. [32]. Future incidence rates of DM were projected from 2010 to 2030, using different scenario analysis to address the rising trend in incidence of DM [31,33,34], as well as the effect of possible interventions that might reduce the incidence of DM.

We estimated annual transition probabilities from undiagnosed to diagnosed diabetes based on the study conducted by Harris et al. [35]. They found that the onset of diabetes occurs, on average, 9–12 years before its clinical diagnosis. Analysis of the proportion of undiagnosed Iranian cases of DM has also revealed that the proportion of undiagnosed cases has decreased by 41% in men and 50% in women between 2005 and 2011 [13]. We assumed that the probability of being diagnosed will increase 5% and 7% per year in men and women, respectively, and a range of 0–10% was considered in sensitivity analysis.

Due to lack of appropriate data regarding diabetes-related mortality in Iranian patients, an age- and sex-specific standardized mortality ratio (SMR) in patients with DM was derived from the study by Tseng et al. in Taiwan [36]. They investigated a cohort of 256,036 diabetic patients with a total 1,124,348 person-years of follow-up to determine the mortality rate, causes of death, and SMR among patients with DM. Synthesis of the literature shows that people with undiagnosed DM have similar mortality rates as people with diagnosed DM [37–39], therefore we assumed the same SMR for people with undiagnosed DM. Different SMR ratios among undiagnosed cases was tested in sensitivity analysis. All input parameters regarding epidemiology of diagnosed and undiagnosed DM are summarized in Table 1.

**Table 1:** Input parameters for the Markov Microsimulation model.

Input variable	Men			Women			Source
	Mean	Min	Max	Mean	Min	Max	
**Diabetes epidemiology**							
**Diagnosed DM Prevalence (%)**							
15–24	0.59	0.19	1.06	0.84	0.33	1.36	[13–15,40,41]
25–34	1.23	0.40	2.21	1.75	0.70	2.83	
35–44	4.24	2.49	6.16	5.96	4.02	7.92	
45–54	11.24	7.66	15.02	15.75	12.24	19.32	
55–64	16.25	12.20	20.29	22.65	19.20	26.15	
**Undiagnosed DM Prevalence (%)**							
15–24	0.64	0.29	1.04	0.88	0.43	1.34	[13–15,40,41]
25–34	1.34	0.61	2.16	1.83	0.90	2.80	
35–44	2.90	1.47	4.54	4.02	2.22	5.85	
45–54	3.08	1.82	4.47	4.29	2.87	5.76	
55–64	4.18	2.62	5.85	5.85	4.22	7.53	
**T2DM Incidence rate per 1000 Person-Years**							
20–29	3.13	1.94	4.79	2.9	1.98	4.1	[31,33]
30–39	7.87	6.11	9.98	7.87	6.34	9.65	
40–49	12.8	10.1	16	14.4	11.9	17.2	
50–59	16.1	12.4	20.5	22.3	18.4	26.8	
60–69	16.7	12.6	21.7	24	18.5	30.5	
70–79	18.2	10.4	29.6	16.9	7.72	32	
>80	21.8	2.64	78.8	21.8	2.64	78.8	
**T1DM Incidence rate per 100000 Person-Years**							
0–4	2.11	1.28	2.94	2.48	1.56	3.4	[32]
5–9	2.68	1.86	3.52	4.44	3.34	5.54	
10–14	4.48	3.38	5.6	5.9	4.6	7.2	
15–19	4.48	1.6	3.54	4.95	3.58	6.32	
20–24	3.29	2.02	4.56	3.42	2.12	4.7	
25–29	3.07	1.76	4.38	3.58	2.14	5.02	
**Standardized Mortality Ratio (SMR) for DM Patients**							
<45	5.33	5	5.67	4.62	4.17	5.11	[36]
45–49	3.94	3.69	4.21	4.52	4.08	4.99	
50–54	3.02	2.86	3.2	3.61	3.34	3.89	
55–59	3.07	2.93	3.21	3.31	3.13	3.5	
60–64	2.51	2.41	2.6	3.01	2.89	3.13	
65–69	2.19	2.12	2.26	2.44	2.36	2.52	
70–74	1.57	1.53	1.62	1.91	1.85	1.97	
> = 75	1.09	1.06	1.11	1.14	1.12	1.16	

### Random-effect Bayesian meta-analysis to estimate mean annual direct and indirect costs

In order to have the most recent information, authors performed a systematic review to identify the cost of diabetes in Iran. The review included all Iranian studies indexed in the international and national databases including Medline, Scopus, Science Direct, Scholar Google, Institute for Scientific Information (ISI), and also Iran Medex and Irandoc for Farsi language papers from 1990 to September 2014. The review was performed with a focus on those studies aimed at measuring incremental cost of DM including direct and indirect costs. The number of cost of illness (COI) studies that reported economic burden of DM including either direct (e.g. cost of inpatients and outpatient health care utilization), indirect (e.g health-related days absent from work (Temporarily Disability), reduced earnings capacity from permanent disabilities and lost productivity from premature mortality), or both types of costs, are presented in Table 2.

After summarizing the estimated average cost of diabetes all costs were converted to US$ and inflated/deflated to 2009 US$ using a consumer price index in Iran [42]. Also estimated mean costs were adjusted with market exchange rates where studies were used an official exchange rate instead of the market price. The estimated mean annual cost of DM ranged from US$226 to US$903 (in 2009 US$).

**Table 2:** Summary of the literature review for cost of DM (all costs are in 2009 US$).

Authors (Year)	Study Design	Sample size	Data	Type of Diabetes	Study perspective	Type of Costs	Average direct cost (2009 US$)	Average indirect cost (2009 US$)
Farshchi et al. (2012)	Retrospective/ Prevalence-based	1000	Individual level data	Type 2	Society	Direct and indirect	520 Male* †490 Female	589 Male* †489 Female
Ghaffari et al. 2009	Cross-sectional/ Prevalence-based	4002	Individual level data	Type 1 & Type 2	Purchasers (patients and insurer)	Direct cost	902.75^††^	-
Javanbakht et al. 2009	Cross-sectional/ Prevalence-based	4500	Individual level data	Type 2	society	Direct and indirect	808.6*	821*
Ezzatabadi et al 2012	cross-sectional/ Prevalence-based	250	Individual level data	Type 2	society	Direct	274*	-
Esteghamati et al. (2004–2005)	Randomized representative/ Prevalence-based	710	Individual level data	Type 1 & Type 2	society	Direct and indirect	226	51.8**
Amini et al. 1998	Prevalence-based	-	Modelling	Type 2	society	Direct and indirect	389.2*	1152*

† Cost were adjusted based on the market exchange rate where official exchange rate were used

†† Total estimated cost for all diabetic patients (2,948,637) was 2661.9 million, yielding a mean annual cost of 902.75 US$

*Adjusted average cost for type 1 and Type 2 DM patient

** Only includes lost work days

To estimate mean annual direct and indirect costs, a random-effect meta-analysis model was fitted using WinBUGS software[43]. A Bayesian approach was used to combine existing knowledge with prior information based on established rules of probability. Prior knowledge can be included into the Bayesian model by choice of an appropriate prior distribution. For this study, a simple random-effects model was fitted:

yi∼N(μi,πi)πi=SSi/(SDi^2)μi∼N(θ,Prec)Prec=1/τ^2θ∼gamma(1,0.0001)τ∼uniform(0,10000)

*As it is a random-effect model*, *study-specific mean cost*, μ_*i*_ *are allowed to be different from each other and are assumed to be sampled from a normal distribution with mean θ and variance of τ; SS and SD represent the sample size and standard deviation for each study*, *i = 1……*.*K*, *and θ is our prior distribution for the mean annual cost with assigned gamma distribution*.

All WinBUGS codes are available upon request. We checked the convergence of the model in different number of iterations and finally 50,000 Markov Chain Monte Carlo (MCMC) iterations after a burn-in period of 10,000 was chosen to get posterior distributions. The convergence was checked graphically using trace and autocorrelation plots. We tried a range of plausible prior distributions for the estimated annual cost and a gamma (1, 0.0001) distribution was found to provide the best performance. All the diagnostic graphs and statistics are provided in the supplementary section (Please see S1 Fig and S2 Fig).

Sex-specific per capita cost was estimated using the ratios reported in studies that provided sex-specific cost information [16]. The estimated average costs were applied in the model for people with diagnosed DM. Evidence suggests that people with undiagnosed diabetes have higher health care cost compared to the general population [44]. Based on the literature we assumed that annual cost of undiagnosed patients is between 26–45% of those with diagnosed DM [44,45]. For projection purpose we assumed that the growth rate of annual per capita health care expenditure to be zero, but a range of between 2%-5% was considered in sensitivity analysis. Estimated mean annual indirect costs were adjusted for those studies which did not include all types of indirect costs in their estimation, using proportion of each type of indirect cost reported in the other COI studies in Iran [16,17,46]. To project future indirect costs 1% real annual growth rate in earnings was assumed and a range of 0–5% was tested in sensitivity analysis.

### Model Validity

To assess validity of the model, the International Society for Pharmacoeconomics and Outcomes Research (ISPOR) task force has recommended four main types of validation including: face, internal, cross and predictive validity [47].

The model structure, all data and their sources and results were presented to an expert group of health professionals to determine face validity. The group was asked to evaluate the model structure and assumptions in comparison to real world circumstances. They also assessed the appropriateness of the data sources. A wide series of sensitivity analysis was performed to evaluate internal validity. We assigned some null and extreme values for input parameters such as zero unit cost or probabilities and ran simulations separately for each validation scenario to compare results with base case values and to test the robustness of the outputs. In addition, we ran 1,000 simulations and investigated the cost profile by age group through the simulation time horizon which helped us to assure that the results accurately reflected real-world conditions.

Cross validity of the model was assessed by comparing the model outputs with observed or estimated outcomes in different studies. We compared the sex-specific number of deaths and immigration predicted by the model with the actual number of deaths reported by national death registry and number of migrations reported by Statistical Center of Iran (SCI) [27]. Predicted diabetes-related deaths were compared with a previous estimate from a comparative risk assessment method developed by WHO for global burden of disease project [48].

Finally, information provided by SuRFNCD in 2005 and 2011 was used to check the external validity of the model. Data on DM prevalence in 2005 as well as DM incidence and relevant mortality data were used to recalibrate the model for 2005. Then we ran the simulation and estimated the number of the people with diabetes after 6 years. The estimated number of people with diabetes was compared with the data provided by SuRFNCD in 2011. There was less than 5% difference between our estimation and SuRFNCD data.

### Sensitivity analysis

Both probabilistic and deterministic sensitivity analysis were conducted to explore parameter(s) and other forms of uncertainty surrounding the model. To conduct probabilistic sensitivity analysis (PSA) all parameters including costs and transition probabilities were defined as statistical distributions in the model. Ranges and distributional assumptions for input parameters were based on the literature and nature of the parameters. We assigned gamma distribution for cost, beta distribution for transition probabilities and log-normal distribution for hazard ratios. Then we ran the model as probabilistic with a generation of 1,000 trials within each patient level simulation, which means for each individual simulation the simulation was repeated 1000 times and each time model parameters were drawn from pre-specified probability distributions.

Deterministic sensitivity analyses were conducted by varying key assumptions and parameters used in the base-case analysis. In brief, we assessed the effect of changing of 10–25% (decrease / increase) in DM incidence and diabetes-related mortality and 20–50% changing in mean annual direct and indirect cost. We also tested effect of applying 1–3% annual change in DM incidence rate over the time and 0–5% change in annual earning and health care expenditure on the estimated outcomes. We changed each of these variables while all other variables were held constant then we ran the simulation and recorded the outcomes. We also conducted best- (i.e. applying low rate of incidence and prevalence of DM, low SMR, high ratio of undiagnosed cases and high per capita direct and indirect cost) and worst-case (i.e. applying high rate of incidence and prevalence of DM, low ratio of undiagnosed cases, high SMR and per capita cost) scenario analysis. All models and simulations were constructed in Treeage Pro 2014 software [49].

### Ethics Statement

Ethics approval was not required.

## Results

### Diabetic population size

**Fig 2:**
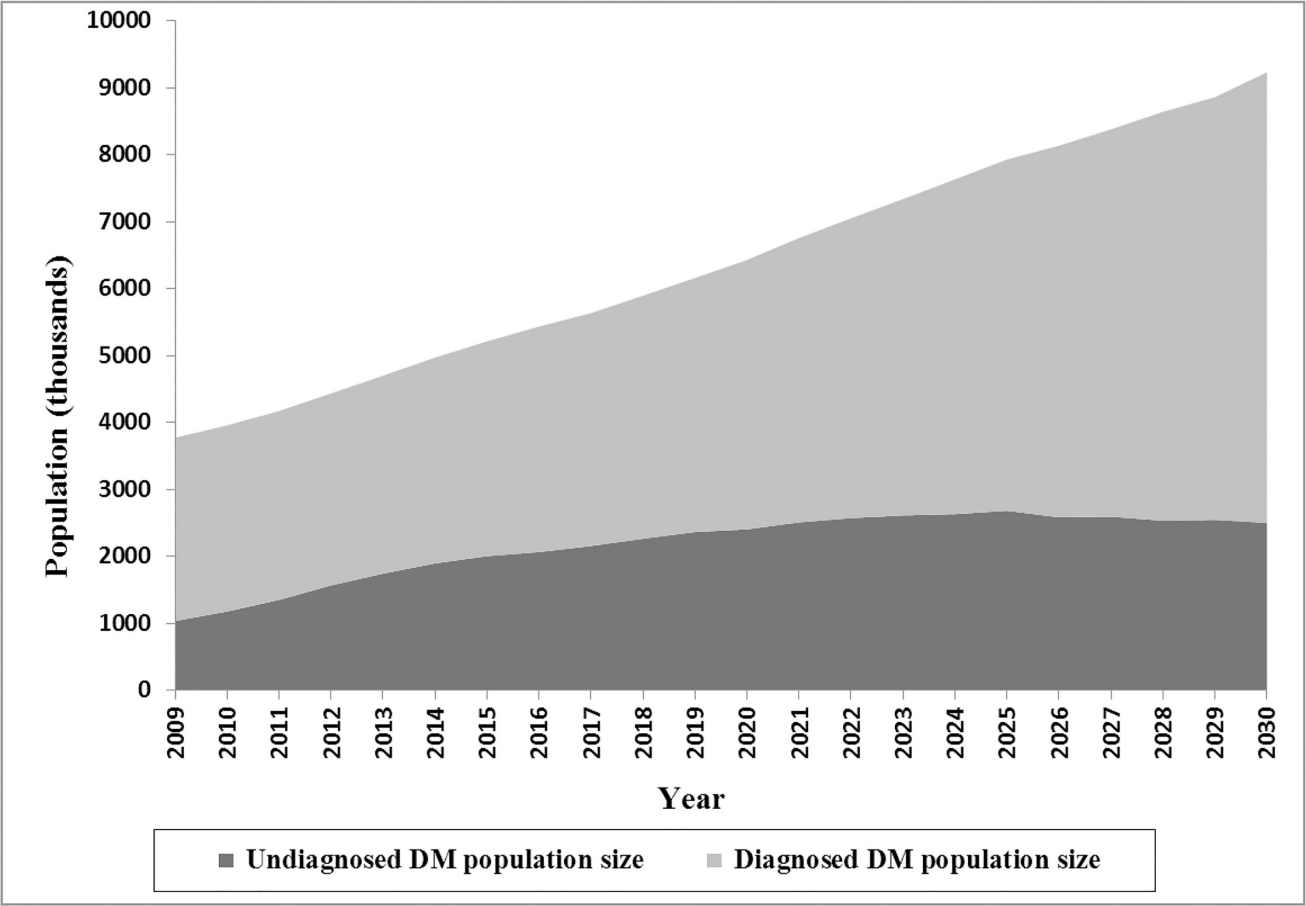
Total estimated diagnosed and undiagnosed DM population size through 2030.

The estimated DM population size is plotted in Fig 2. The model suggested that there were approximately 3.78 million cases of DM in Iran in 2009 (including: 2.74 million diagnosed and 1.04 undiagnosed cases), and 55% were female. Over the next 21 years, the overall population of patients with DM is expected to rise to 9.24 million (6.73 million diagnosed and 2.51 million undiagnosed cases). Total number of the diagnosed cases is projected to grow steadily, while the number of undiagnosed patients predicted to increase steeply through 2025 and then stabilize at around 2.5 million. The model suggested that there was about 38,000 (range 27,000–49,000) diabetes-related death in 2009 (9.3%; range, 6.8–12 of total death) and predicted to increase to 89,000 (60,000–112,000) by 2030 (15% (13–17) of total death).

### Estimated economic burden

**Fig 3:**
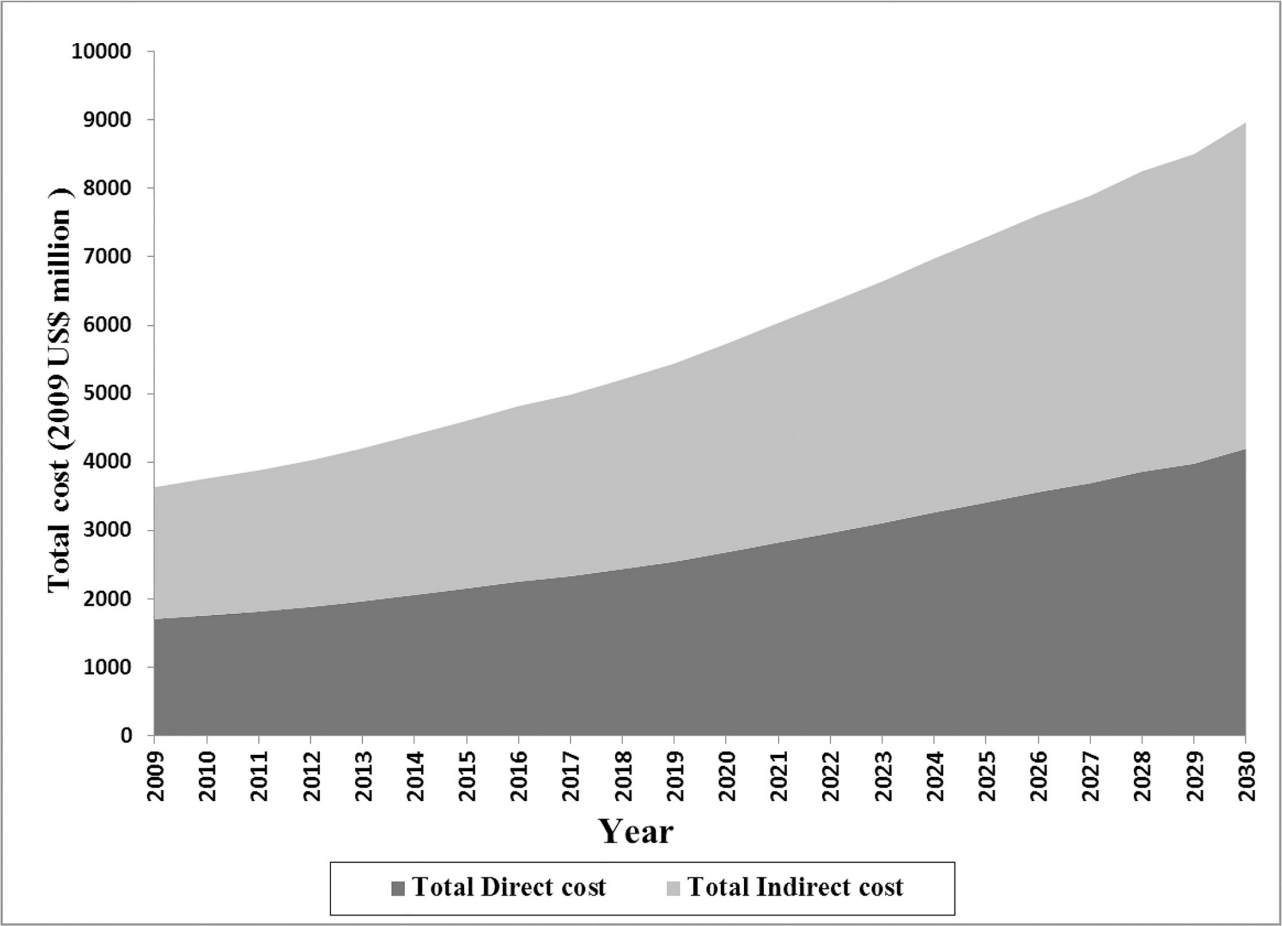
Total estimated direct and indirect cost of DM through 2030.

The Bayesian model indicated that in 2009, the mean annual direct and indirect costs of DM were US$556 (posterior standard deviation [PSD]: 221) and US$689 (PSD: 619), respectively. DM imposed a direct cost of $1.71 billion (2009 US$) on the Iranian health care system, which is equal to about 8% of total healthcare expenditure. Approximately 54% of the expenditure was associated with care for women. In the base-case scenario, direct costs were predicted to rise by 145% through 2030 and reach $4.20 billion (2009 US$). The costs of undiagnosed DM were estimated to account for 11.3% (range, 9.2–14.2) and 11.2% (range, 8.4–13.6) of total direct cost in 2009 and 2030, respectively. The average annual growth rate of direct cost was 4.3%. The pattern of growth in the costs was similar to the growth in DM population size. Total estimated indirect cost is illustrated in Fig 3. Total indirect cost of DM among men and women was estimated to be $1.93 billion (2009 US$) in 2009 and predicted to increase to $4.80 billion (2009 US$) by 2030.

### Sensitivity analysis

**Fig 4:**
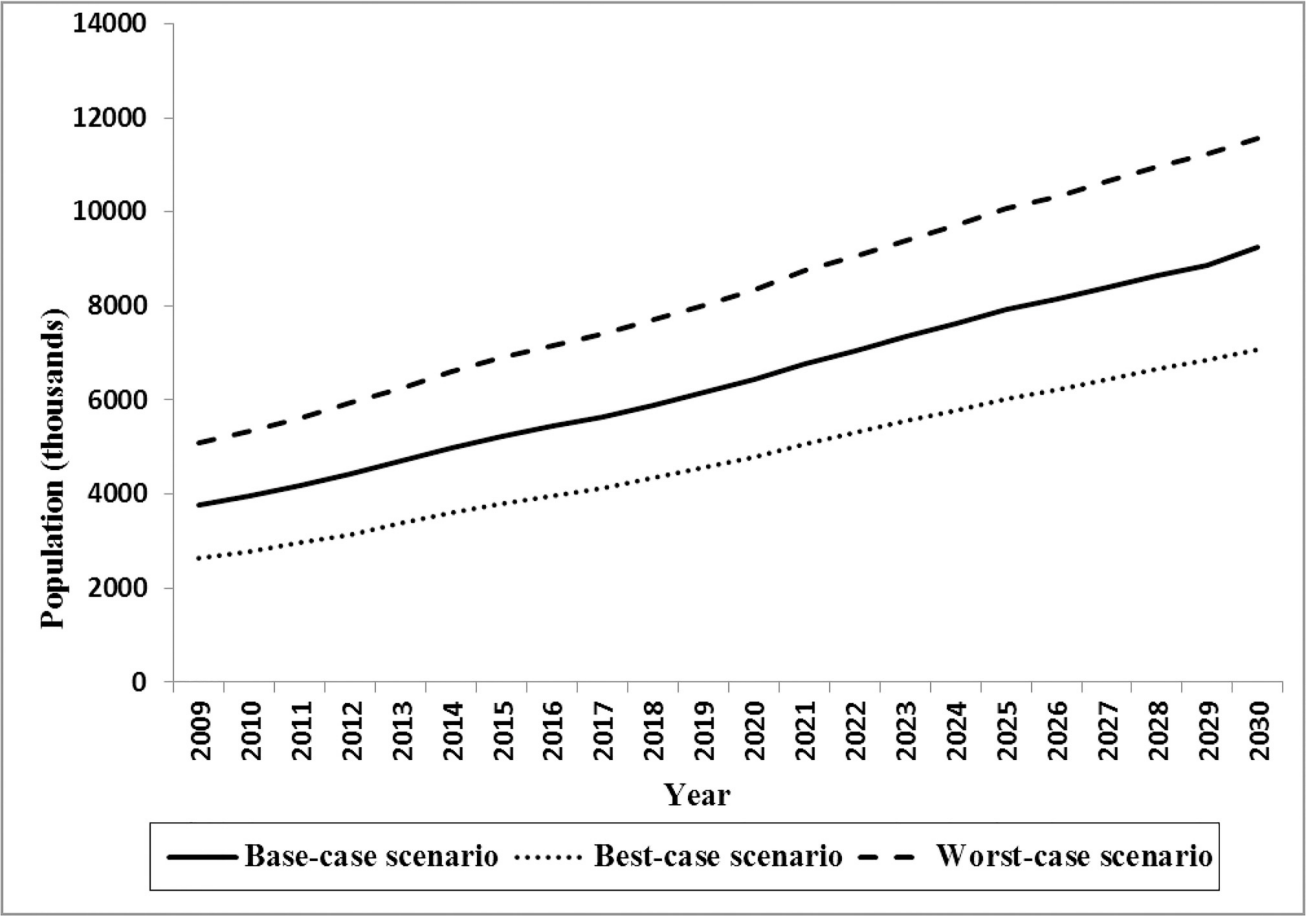
Total estimated DM population size through 2030 in base-, worst- and best-case scenario.

**Fig 5:**
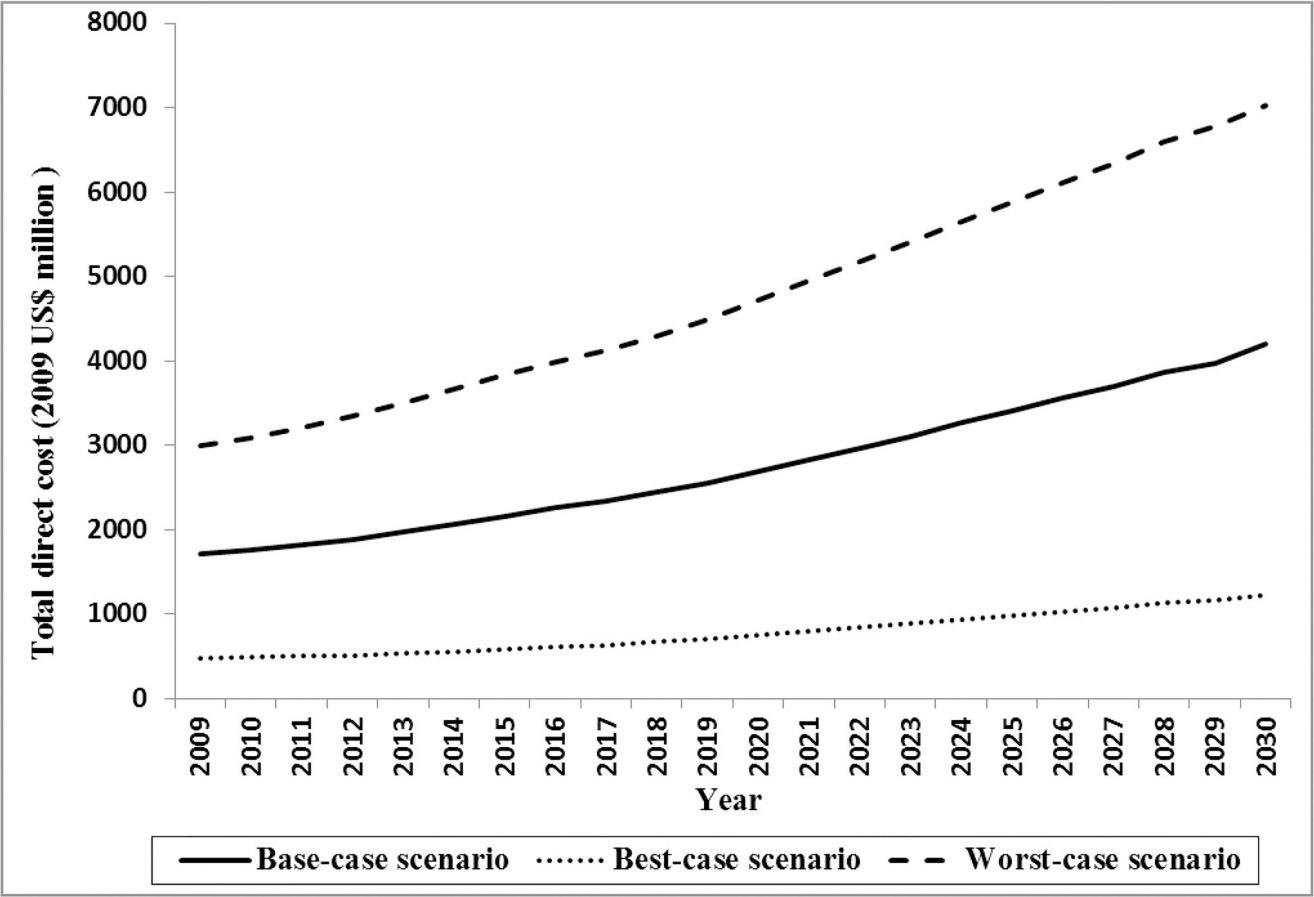
Total estimated direct cost of DM through 2030 in base-, worst- and best-case scenario.

**Fig 6:**
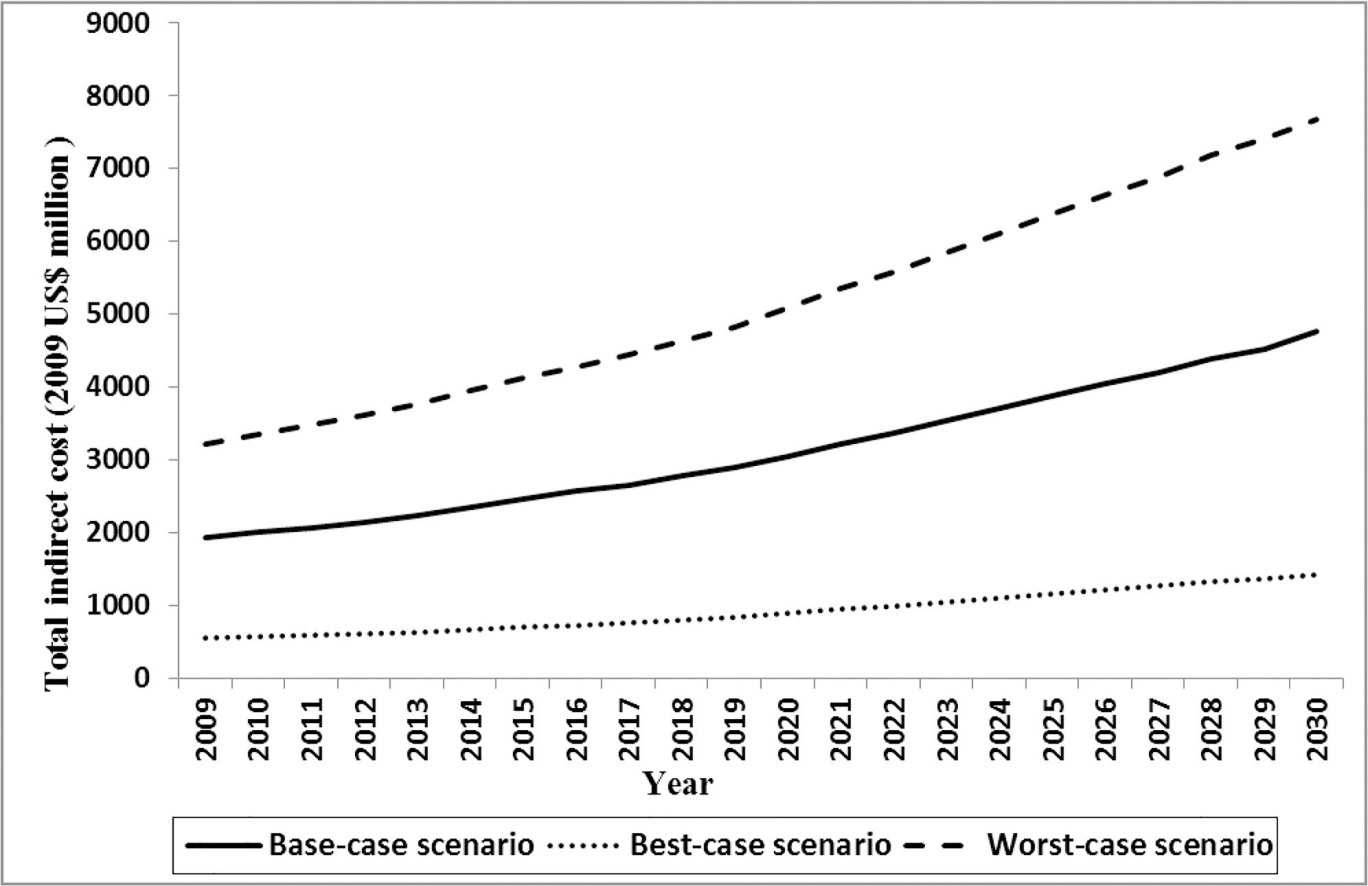
Total estimated indirect cost of DM through 2030 in base-, worst- and best-case scenario.

Based on sensitivity analysis results we found that the total DM population size in best- and worst-case scenario ranged from 2.64 to 5.08 million in 2009 and predicted to increase from 7.82 to 11.55 million by 2030 (Fig 4). Total estimated direct cost in 2009 were US$478 million and US$3 billion (2009 US$) in best- and worst-case scenario and predicted to grow to US$1.22 and US$7.03 billion (2009 US$) by 2030 (Fig 5). The model also showed that the total indirect cost ranged from $548 million to $3.22 billion (2009 US$) in best- and worst-case scenario in 2009 and predicted it would rise to $1.42 and $7.68 billion (2009 US$) by 2030, respectively (Fig 6). One-way sensitivity analysis also revealed that incidence and prevalence of DM and per capita DM related cost were among the input parameters which had the highest effect on the estimated population size and associated economic burden. All results in this section are illustrated in S1 Table.

## Discussion

We projected the number of Iranian patients with diagnosed and undiagnosed DM and its associated economic burden using age- and sex-specific DM data provided by national health surveys and synthesized the existing economic data regarding cost of DM. We used an individual-level simulation model which has been identified as a useful method for predicting trends in health outcomes, projecting the impact of health programs and policies, and comparing efficiency of different health interventions [21]. Such models are particularly effective for addressing complex questions that require synthesis of data from different sources such as projection of healthcare consequences and associated cost of chronic diseases and aging.

This study revealed that about 3.78 million patients with DM were living in Iran in 2009 which is expected to rise to 9.24 million by 2030. This increase in DM prevalence can be explained by increases of urbanization and ageing as well as obesity and being overweight in Iran [50]. Based on our findings the overall prevalence of DM was 5.1% in 2009 and was expected to rise to about 10% by 2030. We found that during the projection horizon, the total number of the diagnosed cases will grow steadily while the number of undiagnosed patients is predicted to increase steeply through 2020 and then stabilize at around 2.5 million. This can be explained by implementation of diabetes prevention and control program in Iran from 2003 which aimed to screen those at risk of DM as well as to raise awareness about DM within the population and provide free access to glucometers [51].

Up to now several studies have been conducted to project population size of DM but almost all of them have focused on developed countries [6,10,52–56]. Huang et al. estimated that the number of people with DM in United States increase from 23.7 to 44.1 million during 2009–2034. They also projected that the number of diagnosed DM patients was predicted to increase while the undiagnosed case steadily declines and stabilizes at 3.7 million by 2020 [56].

Boyle et al. also showed that diabetes prevalence (diagnosed and undiagnosed cases) was predicted to increase from 14% in 2010 to 21% of the US adult population by 2050 [10]. In another study set in the US, Honeycutt et al. showed that the number of people with diagnosed DM increased from 12 to 39 million from 2000 to 2050, corresponding to an increase in prevalence from 4.4% to 9.7% in the total population[55]. Also Waldeyer et al. showed that the number of T2DM will grow from 5 million in 2010 to a maximum of 7.9 million in 2037 in Germany [6]. They concluded that most of the growth is driven by changes in obesity and overweight rates as results of a sedentary lifestyle, increased caloric intake and reduced energy expenditure.

We predicted the trend of annual DM related costs in the Iranian population would start at US$3.64 billion in 2009 (in 2009 US$, including US$1.71 billion direct and US$1.93 billion indirect costs) and would increase to US$9.0 billion (in 2009 US$, including US$4.2 billion direct and US$4.8 billion indirect costs) by 2030. DM related direct and indirect costs were predicted to increase by 129% and 131% between 2009 and 2030, respectively. This increase is attributable to growth in the number of the patients with DM and their life expectancy over the next two decades.

Our estimates for current and future annual direct cost of DM are different from prior efforts by International Diabetes Federation (IDF). The IDF estimated the annual diabetes-related health expenditure worldwide, but it lacked detailed country-specific input data and also used simpler methods to estimate costs in each country. It estimated the costs of both types of diabetes in Iran to be US$1,048–1,829 million in 2010 and US$ 2,186–3,827 million in 2030 [57]. This large variation is due to different methods of analysis; they used the ratio of per capita costs of individuals with diabetes to people without diabetes in the range of 2 to 3. As the estimations based on the ratio of 3 are much closer to our results, it seems assuming a ratio of 2 would cause underestimation of DM costs in Iran. A ratio close of 3 is in agreement with results of the study by Esteghamati et al [18]. They conducted a randomized trial to estimate extra cost of DM in Iran and concluded that ratio of people with DM to those without DM is 2.92. Another explanation for the difference between our estimations and IDF’s is that the IDF assumed a fixed age- and sex-specific diabetes prevalence which may not be a realistic assumption. Prevalence rates change over time when disease dynamics (i.e. incidence and mortality rates) are taken into account. As we’ve shown, the prevalence of DM is expected to rise from 6% to 10% by 2030. Also in contrast to IDF’s study, we assumed different per capita cost for people with undiagnosed diabetes compared to diagnosed DM. Prior studies have shown that the ratio of cost of undiagnosed to diagnosed is 26% in the US [44,45]. Evidence from the literature suggests that there are some differences between estimated DM-related health expenditure by IDF and single studies in other countries too [6,58].

### Limitations

Like with all studies, several limitations exist in this study that should be addressed. Firstly, forecasting DM population size over the next 21 years is fraught with uncertainty. One source of uncertainty arises from incomplete data on age- and sex-specific prevalence, incidence and mortality risk. Due to lack of data in base-case scenario we assumed that the incidence of T2DM in people younger than 20 and incidence of T1DM in people aged 30 and older to be zero. Although due to very low incidence of T1DM [32] and its prevalence (2–5% of total cases of DM) [59] in the Iranian population, we believe relaxing this assumption would not remarkably affect the final results. Second, in the base-case analysis we assumed fixed age- and sex-specific incidence rates over time, however we assessed the effect of increasing and decreasing trend of DM incidence through one-way deterministic sensitivity analysis. Moreover, as time passes in the model, people age and are at greater risk of developing DM, which accounts for the effect of ageing on DM incidence. Third, prediction of future costs and health care utilizations are conditional on current rates of utilization. In our model we used the most current estimates of per capita cost rates for DM. However, per capita cost and health care utilization might change over time, and future changes may influence our results. Fourth, the mean annual per capita indirect cost was estimated using data from studies that used the human capital approach to calculate the cost of lost productivity set in Iran. This approach may overestimate the indirect costs associated with diabetes. Despite these limitations, using local cost and sex- and age-specific epidemiologic data in our analysis has made our results more applicable to local health policy makers.

Last but not least, the development of improved estimates for the cost of DM offers improves the accuracy of baseline estimates. This can help health policy makers determine which policies and programs are cost effective and should be considered when defining a comprehensive and optimum plan of care to control and manage of DM.

## Conclusions

Our findings strongly suggest that the economic burden of DM in Iran is predicted to increase markedly in the coming decades. Much of this burden is preventable through public health initiatives. Identification and implementation of effective strategies to prevent and manage DM should be considered as a public health priority.

## Supporting Information

S1 Fig Bayesian Model results on direct cost (density, autocorrelation and trace graph). (DOCX)

S2 Fig Bayesian Model results on indirect cost (density, autocorrelation and trace graph). (DOCX)

S1 Table Scenario analysis results. (DOCX)
