# Supplementary material for: Projection of Diabetes Population Size and Associated Economic Burden through 2030 in Iran: Evidence from Micro-Simulation Markov Model and Bayesian Meta-Analysis
Source: PLoS One. 2015 Jul 22;10(7):e0132505. doi: 10.1371/journal.pone.0132505 (PMC4511591; doi:10.1371/journal.pone.0132505)
Supplement: S1 Fig — (DOCX) [file pone.0132505.s001.docx]

**S1 Fig.** Bayesian Model results on direct cost (density, autocorrelation and trace graph)
