# Supplementary material for: Projection of Diabetes Population Size and Associated Economic Burden through 2030 in Iran: Evidence from Micro-Simulation Markov Model and Bayesian Meta-Analysis
Source: PLoS One. 2015 Jul 22;10(7):e0132505. doi: 10.1371/journal.pone.0132505 (PMC4511591; doi:10.1371/journal.pone.0132505)
Supplement: S1 Table — (DOCX) [file pone.0132505.s003.docx]

S1 Table Scenario analysis results

| **Scenario** | | **2010** | | | **2020** | | | **2030** | | |
| --- | --- | --- | --- | --- | --- | --- | --- | --- | --- | --- |
|  |  | **DM population size (thousand)** | **Direct Cost (million (2009 US$)** | **Indirect Cost (million (2009 US$)** | **DM population size (thousand)** | **Direct Cost (million (2009 US$)** | **Indirect Cost (million (2009 US$)** | **DM population size (thousand)** | **Direct Cost (million (2009 US$)** | **Indirect Cost (million (2009 US$)** |
| 0 | Base-case analysis | 3962 | 1761 | 2001 | 6434 | 2683 | 3048 | 9236 | 4199 | 4768 |
| 1 | 10% increase in DM Incidence | 3992 | 1768 | 2009 | 6782 | 2798 | 3178 | 9885 | 4482 | 5089 |
| 2 | 25% increase in DM Incidence | 4043 | 1777 | 2018 | 7282 | 2957 | 3359 | 10885 | 4920 | 5586 |
| 3 | 10% decrease in DM Incidence | 3944 | 1758 | 1997 | 6071 | 2557 | 2905 | 8556 | 3895 | 4425 |
| 4 | 25% decrease in DM Incidence | 3876 | 1749 | 1987 | 5518 | 2370 | 2693 | 7522 | 3443 | 3910 |
| 5 | 10% increase in DM related Mortality | 3946 | 1754 | 1993 | 6372 | 2652 | 3012 | 9122 | 4139 | 4700 |
| 6 | 25% increase in DM related Mortality | 3938 | 1751 | 1988 | 6300 | 2620 | 2974 | 8980 | 4063 | 4612 |
| 7 | 10% decrease in DM related Mortality | 3965 | 1762 | 2001 | 6494 | 2709 | 3077 | 9336 | 4248 | 4825 |
| 8 | 25% decrease in DM related Mortality | 3982 | 1771 | 2012 | 6583 | 2753 | 3128 | 9508 | 4338 | 4928 |
| 9 | 10% increase in DM Prevalence | 4370 | 1934 | 2197 | 6714 | 2825 | 3210 | 9410 | 4297 | 4882 |
| 10 | 25% increase in DM Prevalence | 4941 | 2197 | 2496 | 7116 | 3045 | 3459 | 9665 | 4447 | 5052 |
| 11 | 10% decrease in DM Prevalence | 3548 | 1580 | 1794 | 6151 | 2524 | 2866 | 9089 | 4112 | 4669 |
| 12 | 25% decrease in DM Prevalence | 3053 | 1336 | 1516 | 5842 | 2346 | 2664 | 8915 | 4004 | 4547 |
| 13 | 1% increase in annual DM incidence rate | 3958 | 1759 | 1999 | 6661 | 2744 | 3116 | 10120 | 4539 | 5153 |
| 14 | 3% increase in annual DM incidence rate | 3966 | 1763 | 2003 | 7121 | 2878 | 3268 | 11850 | 5220 | 5924 |
| 15 | 1% decrease in annual DM incidence rate | 3969 | 1766 | 2006 | 6211 | 2620 | 2976 | 8354 | 3850 | 4373 |
| 16 | 3% decrease in annual DM incidence rate | 3971 | 1770 | 2011 | 5746 | 2476 | 2814 | 6546 | 3144 | 3574 |
| 17 | 20% increase in mean annual direct and Indirect cost | 3962 | 2114 | 2401 | 6434 | 3220 | 3657 | 9236 | 5038 | 5722 |
| 18 | 50% increase in mean annual direct and Indirect cost | 3962 | 2642 | 3001 | 6434 | 4025 | 4571 | 9236 | 6298 | 7152 |
| 19 | 20% decrease in mean annual direct and Indirect cost | 3962 | 1409 | 1601 | 6434 | 2146 | 2438 | 9236 | 3359 | 3815 |
| 20 | 50% decrease in mean annual direct and Indirect cost | 3962 | 881 | 1000 | 6434 | 1342 | 1524 | 9236 | 2099 | 2384 |
| 21 | 2% increase in annual health care expenditure | 3962 | 1797 | 2001 | 6434 | 3273 | 3349 | 9236 | 5962 | 5712 |
| 22 | 5% increase in annual health care expenditure | 3962 | 1849 | 2001 | 6434 | 4159 | 3349 | 9236 | 8607 | 5712 |
| 23 | 0% increase in annual earnings | 3962 | 1761 | 1961 | 6434 | 2683 | 2685 | 9236 | 4199 | 3730 |
| 24 | 5% increase in annual earnings | 3962 | 1761 | 2080 | 6434 | 2683 | 4677 | 9236 | 4199 | 9678 |
| 25 | 0% annual growth of probability of being diagnosed | 3962 | 1761 | 2001 | 6416 | 2543 | 2886 | 9240 | 3730 | 4230 |
| 26 | 10% annual growth of probability of being diagnosed | 3957 | 1757 | 1995 | 6436 | 2783 | 3164 | 9239 | 4478 | 5090 |
| 27 | SMR Ratio in patients with undiagnosed DM is 50% of those with diagnosed DM | 3973 | 1763 | 2002 | 6538 | 2715 | 3085 | 9400 | 4271 | 4851 |
| 28 | SMR Ratio in patients with undiagnosed DM is 20% of those with diagnosed DM | 3983 | 1766 | 2006 | 6597 | 2737 | 3110 | 9497 | 4314 | 4901 |

**DM:** Diabetes mellitus**, SMR:** Standardized mortality ratio
